# Arctic Small Rodents Have Diverse Diets and Flexible Food Selection

**DOI:** 10.1371/journal.pone.0068128

**Published:** 2013-06-27

**Authors:** Eeva M. Soininen, Virve T. Ravolainen, Kari Anne Bråthen, Nigel G. Yoccoz, Ludovic Gielly, Rolf A. Ims

**Affiliations:** 1 Department of Arctic and Marine Biology, University of Tromsø, Tromsø, Norway; 2 Laboratoire d’ECologie Alpine, Université Joseph Fourier, Grenoble, France; The University of Wollongong, Australia

## Abstract

The ecology of small rodent food selection is poorly understood, as mammalian herbivore food selection theory has mainly been developed by studying ungulates. Especially, the effect of food availability on food selection in natural habitats where a range of food items are available is unknown. We studied diets and selectivity of grey-sided voles (*Myodes rufocanus*) and tundra voles (*Microtus oeconomus*), key herbivores in European tundra ecosystems, using DNA metabarcoding, a novel method enabling taxonomically detailed diet studies. In order to cover the range of food availabilities present in the wild, we employed a large-scale study design for sampling data on food availability and vole diets. Both vole species had ingested a range of plant species and selected particularly forbs and grasses. Grey-sided voles also selected ericoid shrubs and tundra voles willows. Availability of a food item rarely affected its utilization directly, although seasonal changes of diets and selection suggest that these are positively correlated with availability. Moreover, diets and selectivity were affected by availability of alternative food items. These results show that the focal sub-arctic voles have diverse diets and flexible food preferences and rarely compensate low availability of a food item with increased searching effort. Diet diversity itself is likely to be an important trait and has previously been underrated owing to methodological constraints. We suggest that the roles of alternative food item availability and search time limitations for small rodent feeding ecology should be investigated.

**Nomenclature:**

Annotated Checklist of the Panarctic Flora (PAF), Vascular plants. Available at: http://nhm2.uio.no/paf/, accessed 15.6.2012.

## Introduction

Current understanding of mammalian herbivore foraging ecology is mainly based on studies focusing on ungulates; see for example [1], [2] and [3]. Other herbivores with a central role in many ecosystems, such as small rodents, have been less studied. Small rodents are non-ruminant herbivores with fast digestion, invest greatly in reproduction and little in growth, generally have a high risk of predation and are often territorial [4]–[6]. They can therefore be expected to have different nutritional needs and face different trade-offs both physiologically and behaviorally than ungulates. Due to such differences in trade-offs, small rodent functional responses, i.e. the relationship between food intake and food availability [7], are also likely to differ from those developed using ungulates as empirical models [8]. Functional response models can improve the understanding of how herbivores select their food and thus aid in predicting how they may cope with current vegetation changes, as well as how they may themselves affect vegetation. A range of parameters have been suggested to be incorporated into functional response models for herbivores [2], [9], [10]. However, to target those parameters that are important determinants of small rodent functional responses, more exploratory empirical work is required to assess which processes shape their food selection in the wild.

Within a food item category such as plant species or genus, small rodent functional responses to food availability have been studied experimentally [8], [9], [11], [12]. In these studies, food availability has, unavoidably, been found to increase food intake. However, various processes such as handling time, bite size or plant spacing have been shown to have the potential to regulate this relationship [9], [11], [12]. Even though feeding trials using single food items may identify mechanisms that operate in the wild, the value of a food item to an animal is relative to what else is available [13]–[15]. Studies investigating how the availability of alternative food items impacts on consumption of other food items by small rodents are, however, scarce [16]–[18]. These studies experimentally demonstrate that availability of a high-quality food item can reduce the consumption of a low-quality food item. In natural environments, a range of food items of different quality are available and the composition of vegetation may vary greatly. However, small rodent functional responses to the spatially and temporally variable food availability in the setting of complex natural plant communities remain unexplored.

Grey-sided voles (*Myodes rufocanus*) and tundra voles (*Microtus oeconomus*) are among the key herbivores of subarctic tundra ecosystems [19] where they greatly modify tundra vegetation during their cyclic population density peaks [20]–[23]. During recent decades, their cyclic population dynamics have dampened in many areas [24]. While changes in winter climate have mainly been suggested to cause these changes in population dynamics, the role of concurrent vegetation changes is unclear [19], [24]–[26]. However, any evaluation of such bottom-up effects in tundra food webs is severely hampered by the current gaps in knowledge of vole diets and how diet is affected by food availability.

Grey-sided voles are considered to prefer *Vaccinium myrtillus* but to also feed on forbs during summer [27], [28], while tundra voles are considered to feed primarily on monocotyledons, with an increased proportion of *Equisetum* and forbs during the summer [29]–[31]. These generalizations are mostly based on microhistological analysis of ingested material [28]–[31] and observations of feeding signs on vegetation [27]. However, taxonomic resolution of microhistological studies of small rodent diets is limited [32], whereas feeding signs of vegetation give limited information on proportional abundance of different food items in diets.

We analyzed stomach contents of grey-sided voles and tundra voles using DNA metabarcoding [33], [34]. This novel methodology has lately opened new avenues of herbivore diet studies, as it enables analysis of large numbers of samples and identification of the ingested plants at a detailed taxonomic level [32], [35]–[39]. We used a spatially extensive study design, spanning across two river catchment areas and two habitats and sampled vole diets and vegetation composition in common locations over two seasons. Thus, we were able to study the impact of food availability on diets and selectivity both at a taxonomically more detailed level than previous studies and across the range of food availability variation present in natural habitats of voles.

We first compared vole diets to vegetation in order to determine which food plants were selected for. We then investigated how vole diets and selectivity were related to availability of food plants. We analyzed these relationships using both plant families and plant functional groups, and use hereafter the term “food item” to describe any plant group. We predicted that the proportion of any preferred food item in diets would increase with its availability but that it also would be affected by availability of alternative food items. We further predicted that selectivity for a food item would also be affected by availabilities of both the food item in question and alternative food items.

Plant families allowed the most precise taxonomic units for diet and vegetation comparison. Plant functional groups coarsely reflect plant nutrient content and digestibility and allowed grouping of plants according to their presumed nutritional value for herbivores [40], [41]. By analyzing vole feeding habits using both taxonomic and ecological groupings we aimed to both perform a taxonomically detailed analysis of vole diet and to evaluate how the different food item units reflect vole feeding ecology.

## Materials and Methods

### Study Area

**Figure 1:**
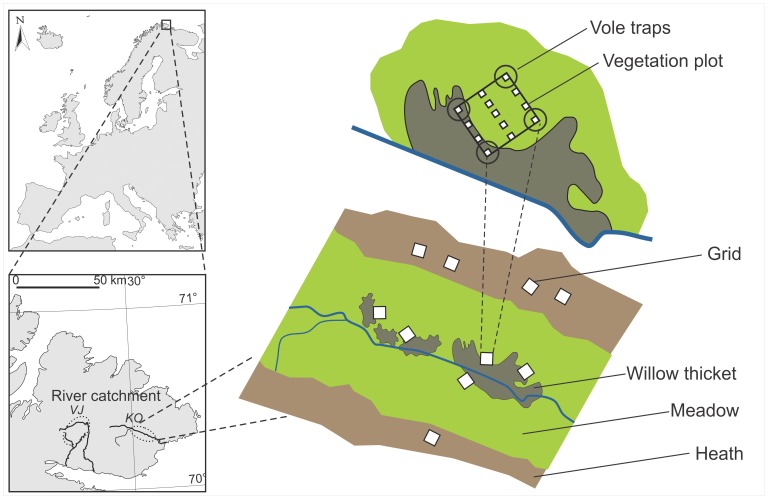
Study location and design. The study was conducted in two river catchment areas; Vestre Jakobselva (VJ) and Komagelva (KO), in low-arctic tundra zone of north-eastern Norway. We established 26 (VJ) and 24 (KO) sampling grids (15 m×15 m), distributed in pairs in heath and meadow habitat throughout major parts of each catchment. In each sampling grid, we estimated plant biomass in 13 plots and small rodent density with 12 traps, 3 per grid corner.

**Figure 2:**
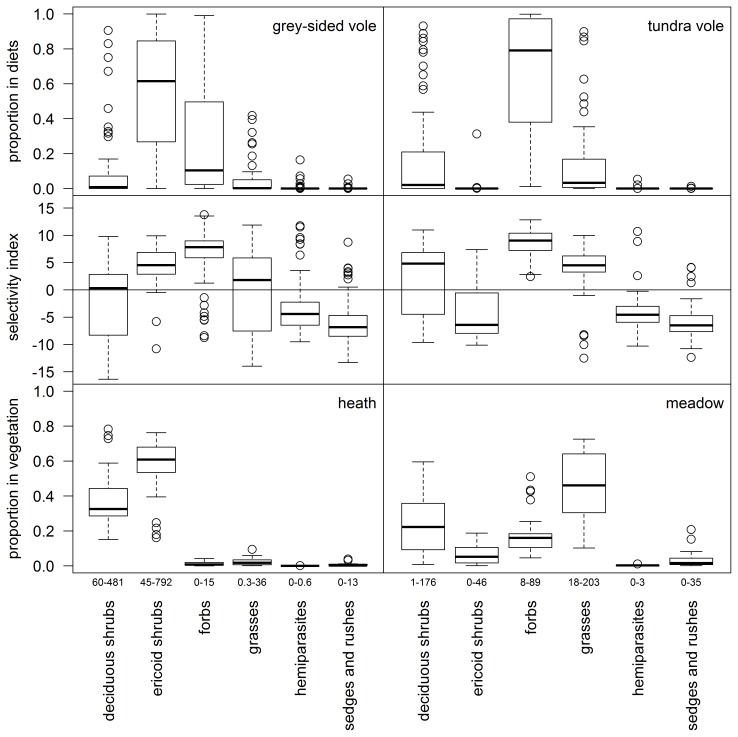
Vole diets, selectivity and food availability based on plant functional groups. To the left grey-sided voles (*Myodes rufocanus*, n = 81) and heath vegetation, to the right tundra voles (*Microtus oeconomus*, n = 66) and meadow vegetation. Upper panels show proportions in diets, middle panels selectivity index and lower panels proportions in vegetation biomass. Selectivity index has been calculated as ratio between diet and vegetation proportions using compositional analysis; see methods for details. Index values above zero indicate preference whereas values below zero avoidance. Black line represents median, boxes first and third quartiles, whiskers either maximum values or 1.5 times interquartile range (whichever is smaller) and points outliers. Numbers below vegetation proportions represent the actual range of biomass (g/m) per group.

This study took place at Varanger peninsula, (70°N, 31°E), Finnmark, North-Eastern Norway (Figure 1). Two prominent habitat types of the area, dwarf-shrub heaths and meadows with scattered willow (*Salix* spp.) thickets harbor different vegetation and small rodent communities. Vegetation in the heath is mostly dominated by *Empetrum nigrum* s. lat. but also *Betula nana* and *Vaccinium myrtillus* are frequent. Field layer of the meadow vegetation is more diverse and dominated by grasses (e.g. *Avenella flexuosa*, *Deschampsia cespitosa*), forbs (e.g. *Rumex acetosa*, *Trollius europaeus*, *Viola* spp.), vascular cryptogams (mainly *Equisetum* spp.), deciduous shrubs (mainly *Salix* spp.), sedges and rushes (e.g. *Carex bigelowii*, *Carex aquatilis* coll., *Juncus filiformis*) and mosses. Average (and range) total plant biomass during this study was 525 g/m (280–1056 g/m) in the heath habitat and 206 g/m (82–439 g/m) in the meadow habitat (see details on biomass measurements below). Biomass ranges for plant functional groups are shown in Figure 2.

In heath habitats, grey-sided voles (*Myodes rufocanus*) are the most common small rodent species, whereas in the meadow habitats tundra voles (*Microtus oeconomus*) dominate the small rodent community [26], [42]. In addition to voles, Norwegian lemmings (*Lemmus lemmus*) are found in the area during their outbreak years. Small rodent populations in the region have cyclic population dynamics with high-amplitude peaks every 4–5 years [43], [44] and this study included a summer season peak in 2007. In addition to small rodents, semi-domesticated reindeer are abundant in the study area, whereas other mammalian herbivores are scarce. More detailed descriptions of the study area can be found in [22], [42], [45] and [45].

### Study Design

In order to cover the range of variation in vegetation composition present at Varanger peninsula, we used a large-scale study design encompassing two river catchment areas; Komagelva (KO) and Vestre Jakobselva (VJ). In both river catchments, we established sampling grids (15×15 m) in equal numbers in both meadows and heaths (Figure 1). The sampling grids were selected to represent the range of variable field layer species compositions of both habitats. In total, KO had 12 sampling grids per habitat and VJ had 13. The distance between neighboring grids had a range of 160–2200 m, while the two most distant grids were 40 km apart. In order to measure food availability in the immediate habitat of each vole individual, we used the same study design for both vole trapping and plant biomass analysis. Population dynamics of voles differ between the focal river catchments [22], [42] and both vole species had lower densities in VJ compared to KO (Table 1).

**Table 1:** Vole density index during 2007 at Varanger peninsula.

	Grey-sided vole		Tundra vole	
	summer	autumn	summer	autumn
Vestre Jakobselva	3.21 (1.95)	8.97 (1.88)	2.56 (0.89)	3.53 (1.14)
Komagelva	7.64 (2.61)	14.58 (2.32)	14.24 (2.92)	27.43 (4.34)

Vole density index during 2007 in two river catchments at Varanger peninsula, based on snap-trapping, measured as individuals per 100 trapnights per sampling grid (mean and SE). Data for grey-sided voles is from heath grids only, for tundra voles meadow grids only. Vestre Jakobselva had 13 sampling grids per habitat, Komagelva 12.

### Vole Trapping; Samples for Diet Analysis

In order to obtain samples for diet analysis, we conducted snap-trapping of voles in each sampling grid according to [46]. To estimate changes in diet during the growing season, the trapping was done twice, with the first period occurring between 22^nd^ and 24^th^ July and the second period occurring between 3^rd^ and 5^th^ September. Each trapping event consisted of 600 trap nights per habitat, with 12 traps in each grid, 25 grids and trapping over 2 nights. The traps were baited with raisins (*Vitis vinifera*) and oat flakes (*Avena sativa*). Voles were dissected and their stomachs stored in 70% ethanol until diet analysis.

Snap-traps were required, as the rodent trapping was part of a project where also the Norwegian lemmings were studied. Norwegian lemmings are hard to trap with live-traps, a phenomenon which has been repeatedly observed by different research groups [47]. In another study using live-traps, only one lemming was caught despite a large trapping effort (ca 6,000 trap-nights every year) and the occurrence of two small rodents peaks (2007 and 2011, resulting >10, 000 trapped voles) (Ims and Yoccoz unpublished data).

### Vegetation Composition; Food Availability Data

Vegetation of each grid was sampled during the peak of the growing season, i.e. between 22^nd^ July and 8^th^ August. We established 13 vegetation sampling plots (0.5×0.5 m) in each grid (Figure 1) and estimated the biomass of all vascular plant species present in the plots using a non-destructive point intercept method [48], [49] with 20 pins in each plot. We then converted the point intercept counts to biomass estimates (g/m) for each grid, by first converting the hits to biomass per plot using calibration described in [50]. In another study across northern Norwegian landscapes, encompassing similar habitats as the current study, plant growth form was found to be the most important predictor for both vegetative and flowering phenology of plants [51]. Hence, the phenology of biomass in our study area could be expected to fairly similar in both river catchements. To account for the temporal changes in biomass we included the effect of season to our analyses.

### Ethics Statements

The study area is part of Varangerhalvøya National Park. No permit was required for the non-destructive vegetation sampling, as only destructive use of vegetation is prohibited in the national park (FOR-2006-12-08-1384, Regulation of Varangerhalvøya nationalpark protection plan). Vole trapping was conducted as part of the “Arctic fox in Finnmark” project (http://www.fjellrev-finnmark.uit.no/), which was initiated, financed and approved by The Norwegian Directorate of Nature Management (DN). The DN is the legal Norwegian authority that licenses sampling of all vertebrate wild life species for scientific purposes (LOV 1981-05-29 nr 38: Lov om jakt og fangst av vilt (viltloven) http://www.lovdata.no/cgi-wift/ldles?doc=/all/nl-19810529-038.html&26) and regulation about sampling wildlife for scientific or other specific purposes (FOR-2003-03-14-349 Forskrift om innfanging og innsamling av vilt for vitenskapelige eller andre særlige formal http://www.lovdata.no/for/sf/md/md-20030314-0349.html). No specific permit was issued for this project, but sampling protocol was approved by the DN. No protected species were sampled.

### Diet Analysis

Stomach contents of grey-sided voles trapped from heath habitat (n = 82) and tundra voles trapped from meadow habitat (n = 67) were analyzed for spermatophyte (i.e. seed plant) content. Part of the dataset is published by [32], who described in detail the DNA metabarcoding methods used (see Table S1 for additional details on the datasets). In summary, spermatophyte plant DNA was amplified from a sample of each voles stomach content using primer pair *g-h*, which targets the P6-loop of chloroplast *trn*L (UAA) intron [52]. Samples from different individuals were thereafter individually tagged, pooled to one sample and pyrosequenced. The resulting sequences were sorted to individual voles based on the tags and compared to two taxonomic reference libraries to identify which taxon they belonged to. We first used a library containing sequences of 842 arctic vascular plants [53] (accession numbers GQ244527 - GQ245667 in GenBank). Thereafter, we compared sequences which could not be satisfactorily identified to sequences retrieved from GenBank (available at http://www.ncbi.nlm.nih.gov/genbank/). For each vole individual, we thus achieved a count of sequences belonging to different taxa. To make data from different vole individuals comparable, we transformed these counts to proportions of different taxa in an individuals’ stomach content, hereafter termed as “diet proportions”.

Quantitative use of DNA metabarcoding data is potentially hampered by several technical issues [38]. However, based on a comparison with traditionally used microhistological method [32], DNA metabarcoding reflects well the actual proportions of spermatophytes in vole diets. We also verified that diet at the vole population level, measured as food item proportions, did not differ greatly from diets determined by frequency of occurrence (i.e. percentage of vole individuals which had ingested the taxa in question), as recommended by [37] (Tables S2 and S3). Moreover, a taxon may be over-represented in a DNA metabarcoding dataset if it has a short target DNA-region in comparison to other simultaneously analyzed taxa [54]. We therefore also confirmed that the most abundant taxa did not have clearly shorter target-DNA regions than other taxa. Both frequencies of occurrence and lengths of the targeted DNA region are given in Tables S2 and S3. We removed two vole individuals from the dataset prior to the analyses. One of these was a grey-sided vole that had seemingly eaten only one plant species, an unlikely result which could easily be due to low DNA quality of the sample. The other was a tundra vole whose diet was composed 99% of *Pinus sylvestris*, a species not present in the study area. Rather than representing a new species in the region’s flora, such a result is probably caused by errors during the analyses [38].

The reference libraries we used included a different range of species than those present in the study area. We therefore checked for potential mis-identifications and adjusted the sequence assignments based on taxa present in Northern Fennoscandia [55]. Taxa which are not found in the region were assigned to their next higher taxonomic level (e.g. *Cerastium maximum* was assigned to *Cerastium* sp. and *Gaylussacia* sp. to Ericaceae). Adjustments were also made to more specific taxa, i.e. when a genus (or family) was represented by only one species (or genus), it was assigned to this representative (e.g. *Bistorta* sp. was assigned to *Bistorta vivipara* and Betulaceae were assigned to *Betula* spp.). Sequences originally assigned to *Vaccinium alaskense*, which is not found in the region were grouped together with those assigned to *Vaccinium myrtillus*. These species are almost identical at the DNA region we used for identification but differ from other *Vaccinium* species of Northern Fennoscandia, namely *Vaccinium uliginosum* and *Vaccinium vitis-ideae* (accession numbers GQ245635-GQ245641 in GenBank).

### Definitions of Food Item Groups

For analysis at plant functional group level we classified plants as forbs, grasses, sedges and rushes, deciduous shrubs, ericoid shrubs, or hemiparasites. The grouping was primarily based on nutritional characteristics [40], [56] as well as responses to herbivory in the focal ecosystem [22], [57]. However, we grouped all ericoid shrubs together as less than half of the sequences within Ericaceae were identified at a detailed enough level to allow distinguishing between deciduous and evergreen shrubs. The deciduous shrubs -group was thus composed of Betulaceae and Salicaceae. Only a few non-ericoid evergreens (Pyrolaceae, Pinaceae, Cupressaceae) were recorded in the diets and each of them occurred only in one vole individual (Tables S2 and S3). These taxa compose a very small fraction of the biomass (on average 0.003% and 0.4% in heaths and meadows respectively) and we therefore excluded them from all analyses. We also excluded data on vascular spore plants (i.e. *Equisetum* and ferns) from the analyses, as the primer pair *g-h* is designed particularly for spermatophytes and does not reflect well the abundance of other plant groups.

For analyses based on taxonomic units, we grouped the plants at family level in order to be able to include majority of the data. For example, 36% of sequences identified to Ericaceae in grey-sided voles diets could not be identified to genera (Tables S2 and S3). However, for two families we had sufficient data to refine the analyses to species level. One of these, Cornaceae is represented in Northern Fennoscandia by only one species (*Chamaepericlymenum suecica*). The other family for which we achieved species level resolution was Ranunculaceae for tundra voles. *Ranunculus acris* coll. was the only representative in the meadow grids and *Ranunculus* sp. constituted 99% of Ranunculaceae in tundra vole diet. We therefore used data on *Ranunculus acris* coll. in all analyses of Ranunculaceae in tundra vole diets and selectivity.

### Statistical Analysis

#### Food selection: compositional analysis

**Figure 3:**
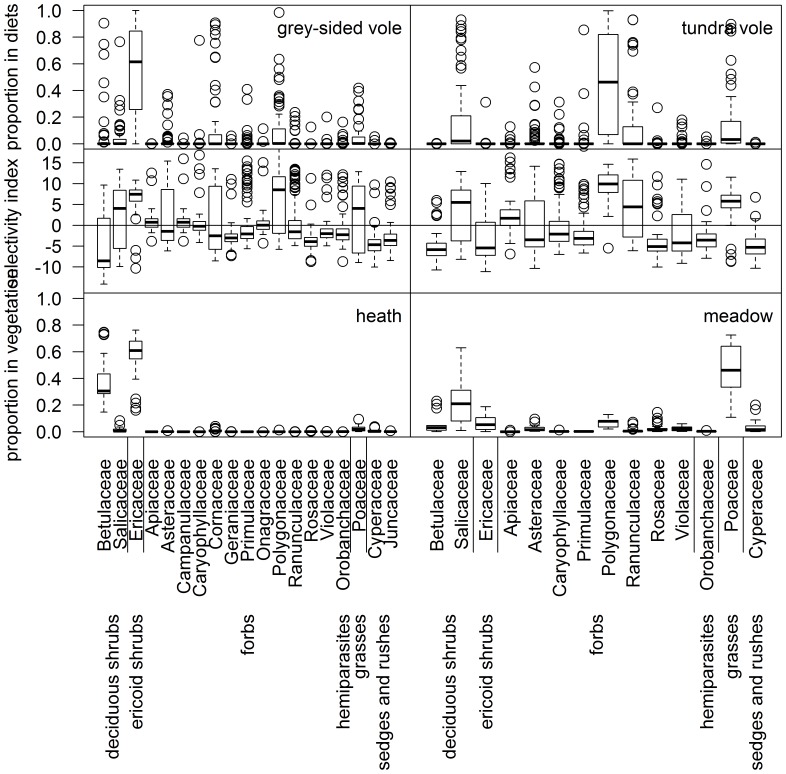
Vole diets, selectivity and food availability based on plant families. To the left grey-sided voles (*Myodes rufocanus*, n = 81) and heath vegetation, to the right tundra voles (*Microtus oeconomus*, n = 66) and meadow vegetation. Upper panels show proportions in diets, middle panels selectivity index and lower panels proportions in vegetation biomass. Selectivity index has been calculated as ratio between diet and vegetation proportions using compositional analysis; see methods for details. Index values above zero indicate preference whereas values below zero avoidance. Black line represents median, boxes first and third quartiles, whiskers either maximum values or 1.5 times interquartile range (whichever is smaller) and points outliers. Plant families are grouped according to functional groups.

To determine selectivity we used compositional analysis of centered log-ratio transformed proportions [58] of food items in individual diets and available vegetation, at both plant family and functional group level. The centered log-ratio transformation was implemented by function named “clr” in in the R library compositions [59]. As food availability data we used for each vole individual, the biomass proportions from the grid in which it was trapped. The selectivity index was calculated as clr(diet proportions) -clr(available proportions) [58]. To test whether selectivity for different food items was significantly different, we used compana -function in adehabitat-library of R, which computes pairwise significances in preference among food items using Wilks lambda [60]. Results of these significance tests are presented in Tables S4, S5, S6, S7, while diets, food availability and selectivity index values are shown in Figures 2 and 3.

Both the data for diet and available food contained zeros, which have to be replaced to enable compositional analysis [58]. We followed recommendations given by [58], replacing zeros with a value three orders of magnitude smaller than any observed used proportion (0.000077) to the diet proportions. This very small replacement value ensured that minute amounts of detectable DNA were not included in the analysis. We excluded plant families which never occurred in diets from the compositional analysis at family level, as their combined biomass was on average <1% in heaths and 2% in meadows. We replaced zero availability in a given grid with average biomass of the food item in question in the same habitat and river catchment. When a food item was not recorded within a river catchment, we used average biomass of the habitat across river catchments. Campanulaceae and Apiaceae were never recorded in the heath habitat, even if they were recorded in the diets of two grey-sided voles. We replaced zero availability in these families by an order of magnitude smaller value than the smallest observed proportion of any family in the heath. We included sequences of trap bite (*Avena* and *Vitis*) in calculations of food item proportions in stomach contents, but excluded them from further analyses.

#### Variability in diets and selectivity: linear mixed effect models

We evaluated whether I) the proportion of a food item in diet and II), selectivity for it were related to vegetation composition using linear mixed effect models implemented by lmer-function from lme-4 library of R [61]. In order to target important food items and retain sufficient sample size for the models, we modeled separately the response of each food item which was both selected for and eaten by at least ca. 50% of the individuals of a given vole species. For grey-sided voles these were the functional group forbs and families Ericaceae, Polygonaceae, Poaceae, Salicaceae and Cornaceae, while for tundra voles they were the functional group forbs and families Polygonaceae, Poaceae, Ranunculaceae and Salicaceae. No other functional group than forbs had several families which were eaten so commonly that they could be tested separately. For all of these food items, we modeled separately proportions in diets and the selectivity index score. For diets, we used logit transformed proportions as the response variable, avoiding zeros by adding a value which was an order of magnitude smaller than the smallest value of the respective predictor variable, while selectivity index scores were already at logit-scale.

For each response variable, we created two alternative models. The first model included as predictors a) biomass of the food item itself and b) biomasses of other substantially eaten food items at family level (i.e. those listed above). However, to better fit data with the voles feeding ecology, we only used biomass of palatable deciduous *Vaccinium* species as predictor instead of Ericaceae. For grey-sided voles, Salicaceae was omitted from models which included Polygonaceae, as their biomasses in vegetation were highly correlated. In the alternative model we replaced the predictor(s) forb families by the forb functional group, leaving the response variable at family level. In all of these models, we evaluated the spatial variability of diets and selectivity in two ways, using river catchment (KO and VJ) as a fixed effect and grid identity as random effect. In addition, we included season (autumn and summer) as a fixed effect. When random effect variance was estimated as zero, we removed the term and present a model with fixed effects only (using lm-function of R). We then selected the better model using likelihood ratio test (model parameters estimated using ML) [62]. When neither model was significantly better, we show the model with less parameters. We present the final mixed effect models with model parameters estimated using REML. In addition to statistically significant effects (defined as 95% confidence intervals not encompassing zero), we included in our interpretation close-to-significant trends which seemed biologically interesting. These are statistically defined as having 95% confidence intervals crossing zero by <0.05 and an effect size of >0.15. After fitting fixed terms of the models, we calculated the proportion of remaining variance explained by random variable “grid identity” (i.e. grid variance/(grid variance+residual variance)) [62].

In each model, we removed those individuals which had a combination of zero availability and zero use of the response food item. We checked models for outliers and removed one heath grid where Polygonaceae biomass was approximately 8 times that of any other grid (thus removing two grey-sided vole individuals). We also verified that models showed constant variance of the residuals and approximate linearity between the fitted and observed values. For models with random effects we estimated significance of the fixed parameters with 95% confidence intervals (Markov Chain Monte Carlo estimation with 100 000 replicates using mcmcsamp -function) [61], while for models without random effects we used the confint-function of R. We used the software R for all analyses [63].

## Results

### Diets

At the level of plant functional groups, diet of the grey-sided voles was dominated by ericoid shrubs, followed by forbs (Figure 2). Deciduous shrubs and grasses were also eaten but less commonly (Figure 2). Within ericoid shrubs, i.e. within the family Ericaceae, deciduous species *Vaccinium uliginosum* (9%) and *Vaccinium myrtillus* (8%) were the most commonly identified but also everegreen shrubs, mainly *Empetrum nigrum* s. lat. (6%) were found (Table S2). Within the functional group of forbs, most abundantly eaten families were Cornaceae (10%, represented by *Chamaepericlymenum suecica*) and Polygonaceae (9%, represented mainly by *Rumex* sp.) (Figure 3, Table S2). Grey-sided voles had also consumed a range of other forb families at a lower proportion, many of which occurred in only a few individuals (Figure 3, Table S2). Species richness of grey-sided voles diet (n = 82 vole individuals) was 28 at species level, 37 at genera level and 23 at family level (Table S2).

At the level of plant functional groups, the diet of tundra voles was markedly dominated by forbs, followed by deciduous shrubs and grasses (Figure 2). The functional group of forbs was dominated by family Polygonaceae (45%, represented mainly by *Rumex* sp.) (Figure 3). While the family Ranunculaceae was also commonly eaten (12%), the mean proportion of other forb families was low and many of them occurred in only a few individuals (Figure 3, Table S3). Species richness of tundra vole diet (n = 67 vole individuals) was 26 at species level, 35 at genera level and 23 at family level (Table S3).

### Selectivity

Both vole species had the strongest selection for forbs (Figure 2, Tables S4 and S5). After forbs, grey-sided voles selected for ericoid shrubs and thereafter grasses, whereas tundra voles selected for grasses and thereafter deciduous shrubs (Figure 2, Tables S4 and S5). The patterns of selectivity at functional group level differed somewhat from those at plant family level. For example, only one forb family, namely Polygonaceae, was more often selected than Poaceae (i.e. grasses), a pattern found for both vole species (Figure 3, Tables S6 and S7). Also within the functional group of deciduous shrubs both vole species showed a similar pattern; Salicaceae was relatively preferred whereas Betulaceae was the least preferred (Figure 3, Tables S6 and S7).

### Spatio-temporal Variation of Diets and Selectivity

Both vole species showed temporal and spatial variation in their feeding habits. During summer, grey-sided voles had higher proportions of Poaceae and forbs, especially Polygonaceae in their diets than during autumn (Table 2). During autumn, they selected more for Ericaceae than during summer (Table 3). Tundra vole diets and selectivity were less modified by season than that of grey-sided voles but tundra voles also selected for Polygonaceae more during summer than autumn (Tables 4 and 5). Spatial variability in diets and selectivity was measured at two scales; river catchment and sampling grid. Of these, river catchment had little effect on the vole diets and selectivity (Tables 2, 3, 4, 5). Only grey-sided voles use of Poaceae varied at the scale of river catchment, with diet proportions and selectivity being higher at VJ than at KO (Tables 2 and 3). However, both vole species showed spatial variability in diet proportions and selectivity at the scale of sampling grids, based on percentage of residual variance explained by grid identity (Tables 2, 3, 4, 5).

**Table 2:** Effect of food availability, season and river catchment on grey-sided vole diets.

Predictors	Responses											
	Polygonaceae (n = 52)		Cornaceae (n = 63)		Ericaceae (n = 79)		Poaceae (n = 79)		Salicaceae (n = 59)		forbs (n = 79)	
	Est.	95% CI	Est.	95% CI	Est.	95% CI	Est.	95% CI	Est.	95% CI	Est.	95% CI
fixed effects												
intercept	−6.01	−7.86, −4.11	−5.89	−8.31, −3.58	0.51	−0.45, 1.47	−9.35	−11.02, −7.47	−6.42	−8.53, −4.51	−3.84	−5.39, −2.14
Polygonaceae	0.11	−6.76, 7.63										
Cornaceae			0.26	−0.27, 0.84	−2.17	−4.41, 0.07						
Poaceae	**0.17**	**0.01, 0.34**	−0.18	−0.45, 0.11	−0.04	−0.11, 0.03	0.04	−0.10, 0.22	−0.03	−0.23, 0.13	0.06	−0.08, 0.21
Salicaceae			*0.23*	*−0.02, 0.53*	−0.01	−0.10, 0.07	0.08	−0.08, 0.25	−0.09	−0.27, 0.07	0.09	−0.08, 0.23
decidious Vaccinium	−0.007	−0.05, 0.03	0.02	−0.04, 0.09	0.01	−0.006, 0.03	0.01	−0.02, 0.04	0.005	−0.02, 0.04	0.005	0.03, 0.03
forbs	*−0.30*	*−0.62, 0.02*	−0.61	−1.61, 0.28			−0.04	−0.36, 0.28	0.02	−0.26, 0.42	0.13	−0.14, 0.44
season (summer)	**3.26**	**0.99, 5.59**	1.06	−1.46, 3.63	−0.90	−2.04, 0.23	**1.91**	**0.10, 4.01**	1.11	−0.57, 3.91	**2.30**	**0.31, 3.90**
river catchment (VJ)	1.28	−1.67, 4.24	−1.95	−6.59, 2.36	0.80	−0.52, 2.12	**3.38**	**0.74, 5.94**	−1.04	−3.86, 1.80	0.23	−2.18, 2.53
random effects												
grid	3.65e−05	(n = 17, <1%)	2.87	(n = 15, 31%)	NA	(n = 19)	1.65	(n = 19, 17%)	1.64	(n = 19, 28%)	1.51	(n = 19, 17%)
residual	3.55		4.23		2.16		3.71		2.65		3.35	

Parameter estimates of linear mixed effect models for the effect of food availability (biomass g/m), season and river catchment on grey-sided vole stomach content proportions. Intercept is calculated for autumn, Komagelva (KO) and mean biomass of all continuous predictor variables. “Est.” refers to regression coefficients, measured at logit-scale. Random effects are presented as standard deviations, sample size (n) referring to the number of grids included in the analysis, % values to the percentage of residual variance assigned to grid. Estimates with bold indicate that 95% CI does not include 0, with italics that 95% CI includes zero at most 0.05 and effect size is >0.15. Models where data were insufficient to evaluate the random effect (NA), have been calculated as linear regressions with fixed effects only. “Forbs” as predictor variable represents availability of the functional group of forbs, except for models which have a forb family (Polygonaceae, Cornaceae) as response variable. For these, biomass of the respective family is excluded from that of forbs and used as a separate predictor. Empty cells indicate that predictor variable in question has not been included in the model. See Material and Methods for details.

**Table 3:** Effect of food availability, season and river catchment on grey-sided vole selectivity.

Predictors	Responses											
	Polygonaceae (n = 51)		Cornacea (n = 61)		Ericaceae (n = 75)		Poaceae (n = 76)		Salicaceae (n = 58)		forbs (n = 77)	
	Est.	95% CI	Est.	95% CI	Est.	95% CI	Est.	95% CI	Est.	95% CI	Est.	95% CI
fixed effects												
intercept	8.34	5.78, 10.90	3.91	0.22, 7.71	7.41	6.66, 8.15	−2.23	−5.54, 1.37	4.14	−1.61, 12.70	4.86	0.66, 9.32
Polygonaceae	−4.93	−14.28, 4.42										
Cornaceae			0.43	−0.39, 1.38								
Poaceae	**0.23**	**0.02, 0.45**	−0.29	−0.73, 0.16	−0.02	−0.09, 0.04	0.03	−0.27, 0.36	−0.08	−0.43, 0.20	0.03	−0.15, 0.23
Salicaceae			0.33	−0.07, 0.79	−0.03	−0.10, 0.04	0.16	−0.17, 0.48	*−0.23*	*−0.54, 0.04*	0.05	−0.16, 0.24
deidious Vaccinium	−0.01	−0.06, 0.04	0.03	−0.07, 0.13	0.004	−0.009, 0.02	0.01	−0.05, 0.08	−0.01	−0.06, 0.06	0.003	−0.03, 0.04
forbs	**−0.58**	**−1.01, −0.16**	−1.02	−2.57, 0.34	−0.04	−0.17, 0.09	−0.03	−0.66, 0.57	0.03	−0.44, 0.74	0.11	−0.28, 0.51
season (summer)	2.39	−0.82, 5.60	1.25	−3.10, 5.42	**−1.12**	**−2.0, −0.22**	3.22	−0.22, 7.32	1.93	−0.88, 6.68	0.63	−1.90, 2.89
river catchment (VJ)	0.16	−3.78, 4.09	−3.00	−10.33, 3.57	0.45	−0.60, 1.51	**5.94**	**0.83, 11.00**	−2.18	−6.79, 3.05	−0.88	−3.95, 2.19
random effects												
grid	NA	(n = 17)	3.97	(n = 15, 24%)	NA	(n = 19)	3.30	(n = 19, 18%)	4.39	(n = 19, 40%)	1.50	(n = 19, 9%)
residual	4.62		7.12		1.68		6.98		5.45		4.58	

Parameter estimates of linear mixed effect models for the effect of food availability (biomass g/m), season and river catchment on grey-sided vole selectivity, i.e. difference between stomach content and biomass proportions. Intercept is calculated for autumn, Komagelva (KO) and mean biomass of all continuous predictor variables. “Est.” refers to regression coefficients, measured at logit-scale. Random effects are presented as standard deviations, sample size (n) referring to the number of grids included in the analysis, % values to the percentage of residual variance assigned to grid. Estimates with bold indicate that 95% CI does not include 0, with italics that 95% CI includes zero at most 0.05 and effect size is >0.15. Models where data were insufficient to evaluate the random effect (NA), have been calculated as linear regressions with fixed effects only. “Forbs” as predictor variable represents availability of the functional group of forbs, except for models which have a forb family (Polygonaceae, Cornaceae) as response variable. For these, biomass of the respective family is excluded from that of forbs and used as a separate predictor. Empty cells indicate that predictor variable in question has not been included in the model. See Material and Methods for details.

**Table 4:** Effect of food availability, season and river catchment on tundra vole diets.

Predictors	Responses									
	Polygonaceae (n = 66)		Ranunculaceae(n = 62)		Poaceae (n = 66)		Salicaceae(n = 66)		forbs (n = 66)	
	Est.	95% CI	Est.	95% CI	Est.	95% CI	Est.	95% CI	Est.	95% CI
fixed effects										
intercept	−1.54	−3.21,0.05	−7.50	−10.08, −4.95	−3.20	−4.51, −1.86	−4.17	−6.48, −1.71	−1.33	−4.41, 1.93
Polygonaceae	0.04	−0.13, 0.21								
Poaceae	0.01	−0.01, 0.04	−0.02	−0.06, 0.03	−0.0004	−0.02, 0.02	−0.02	−0.06, 0.02	0.008	−0.01, 0.03
Salicaceae	−0.01	−0.04, 0.007	0.01	−0.02, 0.04	−0.003	−0.02, 0.01	−0.002	−0.03, 0.03	0.0004	−0.02, 0.02
Ranunculus			0.77	−0.18, 1.72						
forbs	0.01	−0.05, 0.08	−0.05	−0.21, 0.09	−0.02	−0.07, 0.03	−0.05	−0.14, 0.05	0.03	−0.03, 0.08
season (summer)	0.96	−0.67, 2.57	1.17	−1.36, 3.80	−0.47	−1.79, 0.90	−0.12	−2.46, 2.28	0.61	−0.78, 1.99
river catchment (VJ)	2.09	−1.71, 6.03	0.92	−4.98, 6.82	−0.14	−3.06, 2.82	−2.98	−8.72, 2.06	1.50	−1.45, 4.44
random effects										
grid	5.28e−05	(n = 21, <1%)	5.85e−05	(n = 21, <1%)	3.02e-05	(n = 21, <1%)	1.93	(n = 21, 16%)	1.71e-05	(n = 21, <1%)
residual	3.18		4.85		2.64		4.39		2.72	

Parameter estimates of linear mixed effect models for the effect of food availability (biomass g/m), season and river catchment on tundra vole stomach content proportions. Intercept is calculated for autumn, Komagelva (KO) and mean biomass of all continuous predictor variables. “Est.” refers to regression coefficients, measured at logit-scale. Random effects are presented as standard deviations, sample size (n) referring to the number of grids included in the analysis, % values to the percentage of residual variance assigned to grid. Estimates with bold indicate that 95% CI does not include 0, with italics that 95% CI includes zero at most 0.05 and effect size is >0.15. Models where data were insufficient to evaluate the random effect (NA), have been calculated as linear regressions with fixed effects only. “Forbs” as predictor variable represents availability of the functional group of forbs, except for models which have a forb family (Polygonaceae, Ranunculaceae) as response variable. For these, biomass of the respective family is excluded from that of forbs and used as a separate predictor. Empty cells indicate that predictor variable in question has not been included in the model. See Material and Methods for details.

**Table 5:** Effect of food availability, season and river catchment on tundra vole selectivity.

Predictors	Responses									
	Polygonaceae (n = 65)		Ranunculaceae(n = 62)		Poaceae (n = 66)		Salicaceae(n = 66)		forbs (n = 66)	
	Est.	95% CI	Est.	95% CI	Est.	95% CI	Est.	95% CI	Est.	95% CI
fixed effects										
intercept	8.50	7.14, 9.95	3.19	−0.36, 6.75	5.90	4.01, 7.78	4.97	1.73, 8.34	8.10	6.93, 9.26
Polygonaceae	0.04	−0.10, 0.18			*−0.17*	*−0.34, 0.02*				
Poaceae	0.001	−0.02, 0.03	−0.02	−0.08, 0.03	−0.004	−0.03, 0.03	−0.03	−0.09, 0.02	−0.001	−0.02, 0.02
Salicaceae	−0.01	−0.03, 0.007	0.02	−0.02, 0.06	**−0.03**	**−0.06, −0.006**	−0.01	−0.05, 0.03	0.004	−0.01, 0.01
Ranunculus			**1.33**	**0.02, 2.64**	0.20	−0.41, 0.81				
forbs	0.02	−0.04, 0.07	−0.12	−0.32, 0.09			−0.06	−0.18, 0.08	0.04	−0.01, 0.08
season (summer)	**1.99**	**0.56, 3.23**	1.50	−2.19, 5.20	0.41	−1.64, 2.45	−0.56	−3.94, 2.67	1.08	−0.18, 2.17
river catchment (VJ)	0.76	−2.38, 4.25	1.94	−5.99, 9.86	−3.40	−7.54, 0.74	−4.90	−12.95, 1.85	0.75	−1.69, 3.48
random effects										
grid	1.25	(n = 20, 21%)	NA	(n = 21)	NA	(n = 21)	2.25	(n = 21, 12%)	1.31	(n = 21, 28%)
residual	2.41		6.55		3.79		6.19		2.10	

Parameter estimates of linear mixed effect models for the effect of food availability (biomass g/m), season and river catchment on tundra vole selectivity, i.e. difference between stomach content and biomass proportions. Intercept is calculated for autumn, Komagelva (KO) and mean biomass of all continuous predictor variables. “Est.” refers to regression coefficients, measured at logit-scale. Random effects are presented as standard deviations, sample size (n) referring to the number of grids included in the analysis, % values to the percentage of residual variance assigned to grid. Estimates with bold indicate that 95% CI does not include 0, with italics that 95% CI includes zero at most 0.05 and effect size is >0.15. Models where data were insufficient to evaluate the random effect (NA), have been calculated as linear regressions with fixed effects only. “Forbs” as predictor variable represents availability of the functional group of forbs, except for models which have a forb family (Polygonaceae, Ranunculaceae) as response variable. For these, biomass of the respective family is excluded from that of forbs and used as a separate predictor. Empty cells indicate that predictor variable in question has not been included in the model. See Material and Methods for details.

### Impact of Availability on Diets and Selectivity

We found few clear effects of biomass of a food item (i.e. availability) on its use (Tables 2, 3, 4, 5). The sole statistically significant effect was that tundra voles were more selective for Ranunculaceae when its biomass was higher (Table 5). In addition we found one non-significant trend whereby grey-sided voles’ selectivity for Salicaceae decreased with its biomass (Table 3). However, use of several food items decreased with the availability of alternative food items (Tables 2, 3, 4, 5). For grey-sided voles, selectivity for Polygonaceae decreased with biomass of other forbs and Polygonaceae proportion in diets had a similar trend (Tables 2 and 3). Moreover, increasing biomass of Salicaceae tended to decrease the proportion of Cornaceae in the diets of grey-sided voles (Table 2). For tundra voles, selectivity for Poaceae decreased when biomass of Salicaceae increased and had a similar trend with biomass of Polygonaceae (Table 5). We also found opposite patterns in grey-sided voles, i.e. use of a food item increasing with the availability of alternative food items (Tables 2 and 3). Of these, both diet proportions and selectivity for Polygonaceae increased when biomass of Poaceae increased, whereas diet proportions of Cornaceae increased with biomass of Salicaceae (Tables 2 and 3). Some of these indirect effects, both negative and positive ones, were caused by changes in the biomass of food items which were on average less selected for than the response food item.

The use of different forb families responded differently to their respective biomass, biomass of alternative food items and season (Tables 2, 3, 4, 5). However, combined biomass of forbs better predicted the consumption of other food items than those of separate forb families. Only the selectivity of tundra voles for Poaceae was slightly better predicted by a model which included biomass of Polygonaceae and Ranunculaceae as predictor variables than with a model using combined forb biomass ( = 3.62, d.f. = 1, p = 0.06).

## Discussion

Both grey-sided voles and tundra voles consumed a diverse range of food items. Although diets and selectivity varied seasonally and spatially, the biomass of a food item had little effect on its use but sometimes influenced the use of other food items. Together, these results show that both vole species exhibit flexible feeding ecology.

### New Insights into Vole Diets

Most studies on the interactions between grey-sided voles and vegetation have focused on *Vaccinium myrtillus*, which has been considered as the most important food item of this species [64]–[68]. However, our results show that during the snow-free period the species has a diverse diet which includes, in addition to *V. myrtillus*, a range of different herbaceous food items. We also found surprisingly much *V. uliginosum* (on average 9% of diets, eaten by 50% of individuals), even if it is relatively rare in the heath vegetation (2% of biomass in average), indicating that it is selected much more than previously observed (similar numbers for *V. myrtillus* are 8% in diets, eaten by 68% of individuals, 20% of biomass in average). Interestingly, *V. uliginosum* and *E. nigrum* have been suggested to be unpreferred species and eaten only when population densities are high [69]. Further, they have been suggested to produce toxins and therefore to have a negative impact on vole population growth rate even if they constitute only a small proportion of diets [69]. We found additional support for this hypothesis for *V. uliginosum*, which inclusion in the diets of grey-sided voles increased with population density. *V. uliginosum* was found in 25% of the individuals in VJ during summer while corresponding values were 42%, 45%, 62% for VJ autumn, KO summer, KO autumn, respectively (in comparison to population density index in Table 1). For *E. nigrum*, on the other hand, we found no such pattern (proportion of individuals that had ingested it, in same order as above, were 70%, 34%, 50% and 75%). Still, as we observed both of these plant species to be eaten by more than half of the studied grey-sided voles during a peak year, it is possible that these food items play a role in the population dynamics of this vole species at the Varanger Peninsula.

For tundra voles, we found forbs to dominate diets and be highly selected for, unlike previous microhistological stomach content studies which have emphasized the use of especially *Eriophorum* and *Equisetum* [29]–[31]. Such discrepancies between studies are probably partly due to differences in availability resulting in different diets. For example, forbs were abundant and *Eriophorum* absent in our study grids, whereas forbs were rare and *Eriophorum* abundant in habitats where tundra vole diets have been studied before [29]–[31]. Moreover, in similar habitats the closely related field voles (*Microtus agrestis*) have also been found to have diets dominated by dicotyledons [70], and in a cafeteria-test tundra voles showed a preference for forbs [71]. As *Eriophorum* was not included in that test, it remains unclear whether plant availability modifies only vole diets or also preferences. In addition, different methodology may contribute to differences in results. While results based on microhistological methods have a tendency to overestimate monocots, the DNA metabarcoding method used in this study possibly underestimates *Equisetum* [32], [52], [72]. In spite of such methodological discrepancies, habitat-specific food availability is likely to be an important determinant of tundra vole diets.

Both vole species selected for highly palatable functional groups, i.e. forbs and grasses, indicating that vole food preferences are related to plant nutritional quality [41], [56], [73]. However, within plant functional groups different families and species were eaten and selected to a very different degree. For example, some forb species were rarely eaten even if their availability did not greatly differ from that of other, more commonly consumed species. Moreover, the use of forb families responded differently to biomass and season. Different nutritional value may explain such differences but because only few measurements of energy, nutrients or secondary metabolites in subarctic forb species exist [64], [74], [75], we cannot judge the importance of different nutritional characteristics for voles. Within the functional group deciduous shrubs, both vole species preferred willows (*Salix* spp.) but avoided birches (*Betula* spp.), a pattern consistent with palatability of these taxa as well as previous food selection studies of voles [76], [77]. Thus, more detailed patterns of food quality than those reflected by plant functional groups, as defined in this study, seem to direct food preferences of voles. However, field measurements of detailed food-selection units have limitations especially when the food items are scarce. For example, grey-sided voles preferred forbs as a functional group even though at family level most forbs were seemingly not preferred, a pattern which could simply be due to different forbs being available to different individuals. Plant functional groups have mainly been studied from a plant ecological perspective [40], [41], [56], [73] and only few attempts have been made to evaluate them based on herbivores ecology [22], [78], [79]. However, small rodent food-selection units may be best reflected by plant functional groups defined from a herbivores perspective.

Previous analyses of diets of small rodents have used methods that are constrained to a taxonomically coarse resolution. Using DNA metabarcoding we were able to reveal that both vole species had remarkably diverse diets in terms of consuming a large number of plant taxa. In fact, diet diversity as such may be an important attribute of vole diets, as it is in general acknowledged to be an important determinant of herbivore performance [15], [80]. Accordingly, [81] found that species richness of vascular plants in the sub-arctic habitats of grey-sided voles was the most important predictor of female reproductive success. It therefore seems likely, that increased understanding of the role of food item diversity for small rodents should reveal previously unknown aspects of vegetation-small rodent interactions.

### Small Rodent Functional Responses

Several earlier studies on mammalian herbivore food selection have indicated that availability, both absolute and relative of a preferred food item may increase its use [13], [82]. Seasonal effects were common even though we found that the biomass of a food item had little effect on its consumption. Nutrient content of herbaceous plants decreases towards the end of the growing season [73]–[75], [83]. Moreover, while berries produced by ericoid shrubs are more palatable than leaves of these plants they are available only in the autumn [51]. The seasonal changes in grey-sided voles feeding habits, i.e. the decrease of forbs and grasses in diets and increased selectivity for Ericaceae from summer to autumn, thus seem to be related to availability of good-quality food. However, at the resolution of our data, season was best seen as qualitative “index” of changing availability of good-quality food. In addition to availability of a food item, availability of alternative good-quality food items may modify consumption by voles [18]. Our results indicate that a food item which has such indirect effects does not have to be more preferred at the population level. For example, tundra voles selected less for grasses (Poaceae) when the biomass of willows (Salicaceae) increased, even though at population level they preferred grasses to willows. We therefore suggest that the effect of alternative food item availability for small rodent functional responses should be further evaluated. Moreover, based on the seasonal changes of voles’ diets and selectivity, relative differences of nutritional quality between different food items are probably important determinants of such effects.

The spatial effects we found, i.e. voles from the same study grids having more similar diets and preferences than those from different grids, suggest that voles from same local environment are more likely to make similar food choices than voles from different environments. In addition to food characteristics, small rodent feeding habits can be affected by competition and predation risk [6] which could therefore contribute to spatial variation in feeding habits. Vole population densities, especially those of tundra voles, differed drastically between the river catchments (Table 1). However, diets and selectivity differed little between river catchments and therefore intraspecific competition seems unlikely to have caused the spatial patterns we observed at grid level. On the other hand, population density of Norwegian lemmings was higher in VJ where vole densities were lower (Table 1) [22], [84]. Interspecific competition with lemmings may therefore have masked some of the effects of intraspecific competition on vole diets. That food biomass had little effect on diets and selectivity does not support the idea that the spatial effects would be caused by food availability either. Still, food item biomass may not necessarily reflect all vegetation characteristics which are important for determining vole feeding habits. For example, a plant species’ nutritional quality may vary, both temporally, spatially and also between plant parts [83]–[85]. Moreover, positive responses of vole selectivity on availability, evaluated via responses to biomass and season, suggest that voles do not compensate low availability with increased selectivity. This in turn indicates that voles invested little effort in searching and selecting the most preferred food. It is well established that perceived predation risk reduces time herbivores, including small rodents, spend foraging in dangerous habitats [6], [86]. Nevertheless, the interplay between food availability and perceived predation risk, ‘the landscapes of food and fear’, remains poorly understood [86], [87]. In tundra habitats vegetation cover is generally low and predation risk high, especially during small rodent population-peak years [88]. Flexible feeding habits of voles could thus at least partly be an adaptation to minimize time spent searching for food, as emphasized by [89] and [90]. The spatial variation of diets and selectivity which we observed are therefore probably caused by a combination of local vegetation characteristics and search time limitations due to predation risk. Both plant quality and search time limitations have been included in some functional response models for herbivores [2], [10] and we suggest that examining the roles of these parameters for small rodent functional response models should be attempted.

While we here show that the population level patterns in feeding habits of voles are flexible, it is possible that vole individuals are more conservative. At least some of the changes in vole diets are related to changes in gut morphology [91], [92], indicating that individual voles may have physiological limitations related to switching quickly between highly different diets. However, little is known about the flexibility of vole diets at individual level, and it is unclear how fast and drastically individual voles may change their diets.

### Methodological Considerations

While few of the observed effects of food item biomass on stomach content were statistically strong, we are confident that these patterns indeed reflect the relationships between voles and their food. The methods we used to estimate food item use and availability, i.e. stomach contents and biomass of plants, have certain shortcomings. Most importantly, food passes quickly through the digestive system of voles [5] and stomach contents therefore give a snapshot of the vole diet during the last hour. In addition, food item availability to voles may be poorly represented by average g/m biomass of plant species. For example, some food items might reach a height which makes them unavailable for small-statured herbivores like voles and hence average biomass may differ from what is available for voles. Finally, we measured plant biomass during the peak of growing season but sampled vole diets during early and late growing season. Due to seasonal increase of biomass, this may have led to underestimation of selectivity in the summer in comparison to the autumn. However, the only seasonal increase of selectivity was that of grey-sided voles for Ericaceae, which can be well explained by an increase in the availability of berries. That we were able to relate patterns of diets and selectivity to patterns of food availability, such as the biomass of alternative food items or the seasonal changes in availability, in spite of biological and technical noise in the data indicates that those patterns are probably stronger in reality than suggested by our analyses. This explanation is supported by the difference between grey-sided voles and tundra voles, as the sample size was higher and the observed patterns both more abundant and statistically stronger for grey-sided voles. We therefore recommend a larger sample size and a more adapted way of measuring food availability for future studies on small rodent functional responses. Such larger sample size could be achieved by analyses of fecal samples, to avoid lethal methods. For example, DNA metabarcoding coupled with radiotelemetry could provide repeated individual-level data on diets, together with targeted locations for food availability estimates. Such estimates could be achieved by adjusting the point intercept method to better represent the vegetation actually available for small rodents, by for example counting only hits up to 10 cm from ground level and separating between leaves and woody plant parts.

### Conclusions

We conclude that voles have diverse diets and flexible food preferences. Thus, viewing food preferences as a fixed ranking of a few species is likely to be insufficient for understanding small rodent feeding ecology. Diet diversity as such may be a functional trait of small rodent diets that previously has been underrated in the literature because of methodological constrains. Moreover, our results suggest that in order to understand small rodent functional responses, the roles of alternative food items and search time limitations should be further investigated.

## Supporting Information

Table S1 Details of the DNA metabarcoding methodology used, in order of execution, for the two datasets combined for this study. Notes: ^1^Soininen et al. (2009), ^2^available at http://www.grenoble.prabi.fr/trac/ecoPCR/, ^3^available at http://www.grenoble.prabi.fr/trac/OBITools/, ^4^in the final dataset used for analyses.
(DOCX)

Table S2 Diet of Grey-sided voles (n = 82), in heath habitat at Komagelva and Vestre Jakobselva, Varanger peninsula, during summer and autumn 2007. Mean proportion with standard error and frequency of occurrence (percentage of individuals where taxa present). Abundance of taxa at species level is included in the genera, which are included in families. Column “length *g-h*” refers to the length of the DNA region amplified with primer pair *g-h*, based on Sønstebø et al. (2010).
(DOCX)

Table S3 Diet of Tundra voles (n = 67), in meadow habitat at Komagelva and Vestre Jakobselva, Varanger peninsula, during summer and autumn 2007. Mean proportion with standard error and frequency of occurrence (percentage of individuals where taxa present). Abundance of taxa at species level is included in the genera, which are included in families. Column “length *g-h*” refers to the length of the DNA region amplified with primer pair *g-h*, based on Sønstebø et al. (2010).
(DOCX)

Table S4 Grey-sided vole (n = 81) selectivity at plant functional group level, based on compositional analysis comparing used (plant DNA in individuals diet) against available (biomass of grid where the individual was trapped). The table is read along the rows; “+” indicates that food item on a row was used more than that in a column, “−” that it was less used. Tripled sign indicates significant differences. “dec.” refers to deciduous.
(DOCX)

Table S5 Tundra vole (n = 66) selectivity at plant functional group level, based on compositional analysis comparing used (plant DNA in individuals diet) against available (biomass of grid where the individual was trapped). The table is read along the rows; “+” indicates that food item on a row was used more than that in a column, “−” that it was less used. Tripled sign indicates significant differences. “dec.” refers to deciduous.
(DOCX)

Table S6 Grey-sided vole vole (n = 81) selectivity at plant family level, based on compositional analysis comparing used (plant DNA in individuals diet) against available (biomass of grid where the individual was trapped). The table is read along the rows; “+” indicates that food item on a row was used more than that in a column, “−” that it was less used. Tripled sign indicates significant differences. Columns are labeled with abbreviated family names using only the first three letters, rows are labeled with full names in the same order.
(DOCX)

Table S7 Tundra vole (n = 66) selectivity at plant family level, based on compositional analysis comparing used (plant DNA in individuals diet) against available (biomass of grid where the individual was trapped). The table is read along the rows; “+” indicates that food item on a row was used more than that in a column, “−” that it was less used. Tripled sign indicates significant differences. Columns are labeled with abbreviated family names using only the first three letters, rows are labeled with full names in the same order.
(DOCX)
