# Supplementary material for: Arctic Small Rodents Have Diverse Diets and Flexible Food Selection
Source: PLoS One. 2013 Jun 27;8(6):e68128. doi: 10.1371/journal.pone.0068128 (PMC3694920; doi:10.1371/journal.pone.0068128)
Supplement: Table S1 — Details of the DNA metabarcoding methodology used, in order of execution, for the two datasets combined for this study. Notes: 1Soininen et al. (2009), 2available at http://www.grenoble.prabi.fr/trac/ecoPCR/, 3available at http://www.grenoble.prabi.fr/trac/OBITools/, 4in the final dataset used for analyses. (DOCX) [file pone.0068128.s001.docx]

| phase of analyse | published dataset^1^ | additional samples |
| --- | --- | --- |
| samples |  |  |
| grey-sided voles | 28 | 54 |
| tundra voles | 17 | 50 |
| samples per individual | 2 | 1 |
| size of one sample | 10mg | 10-70mg |
| sample processing |  |  |
| DNA extraction | DNeasy Tissue Kit | |
|  | (Qiagen GmbH) | |
| tags for individual identification | 5bp at 5’ end | |
| PCR details published in | Soininen et al. (2009) | |
| DNA purification | MinElute PCR purification kit | |
|  | (Qiagen GmbH) | |
| DNA quantification | BioAnalyzer | |
|  | (Agilent Technologies Inc.) | |
| sequencer | 454 GS FLX | |
|  | (Roche Diagnostics) | |
| sequence annotation |  |  |
| n errors allowed: tag | none | |
| n errors allowed: primer | two | |
| rare sequences removal treshold | <4 | |
| annotation similarity treshold with reference | ≤98% | |
| software sequence retrieval from GenBank | ecoPCR^2^ | |
| software sequence annotation | ecoTag^3^ | |
| n sequences per individual, mean(sd) ^4^ | 991(542) | 1592(1356) |
