# Supplementary material for: Arctic Small Rodents Have Diverse Diets and Flexible Food Selection
Source: PLoS One. 2013 Jun 27;8(6):e68128. doi: 10.1371/journal.pone.0068128 (PMC3694920; doi:10.1371/journal.pone.0068128)
Supplement: Table S2 — Diet of Grey-sided voles (n = 82), in heath habitat at Komagelva and Vestre Jakobselva, Varanger peninsula, during summer and autumn 2007. Mean proportion with standard error and frequency of occurrence (percentage of individuals where taxa present). Abundance of taxa at species level is included in the genera, which are included in families. Column “length g-h” refers to the length of the DNA region amplified with primer pair g-h, based on Sønstebø et al. (2010). (DOCX) [file pone.0068128.s002.docx]

| Family | Genus | Species | mean | SE | frequency | length *g-h* |
| --- | --- | --- | --- | --- | --- | --- |
| Ericaceae |  |  | .55 | .07 | 95 | 45-55 |
|  | *Vaccinium* |  | .35 | .05 | 95 | 37-50 |
|  |  | *Vaccinium uliginosum* | .09 | .04 | 50 | 45 |
|  |  | *Vaccinium myrtillus* | .08 | .02 | 68 | 48 |
|  |  | *Vaccinium vitis-idaea* | .008 | .006 | 29 | 50 |
|  | *Empetrum* | *Empetrum nigrum* | .06 | .03 | 55 | 46 |
|  | *Arctous* | *Arctous alpina* | .001 | .002 | 6 | 48 |
|  | *Oxycoccus* | *Oxycoccus microcarpus* | .0002 | .0003 | 1 | 46 |
|  | *Andromeda* | *Andromeda polifolia* | .0001 | .0002 | 1 | 45 |
|  | *Kalmia* | *Kalmia procumbens* | .00003 | .00007 | 1 | 51 |
| Cornaceae | *Chamaepericlymenum* | *Chamaepericlymenum suecicum* | .10 | .05 | 48 | 50 |
| Polygonaceae |  |  | .09 | .04 | 56 | 27-32 |
|  | *Rumex* |  | .06 | .03 | 51 | 31 |
|  | *Bistorta* | *Bistorta vivipara* | .03 | .02 | 21 | 32 |
| Betulaceae | *Betula* |  | .04 | .03 | 33 | 61 |
| Poaceae |  |  | .04 | .02 | 56 | 48-58 |
|  | *Phleum* | *Phleum alpinum* | .02 | .009 | 33 | 53 |
|  | *Avenella* | *Avenella flexuosa* | .008 | .009 | 26 | 53 |
|  | *Deschampsia* | *Deschampsia cespitosa* | .003 | .006 | 2 | 53 |
|  | *Poa* |  | .0004 | .0006 | 10 | 53 |
|  | *Festuca* |  | .0003 | .0004 | 10 | 53 |
| Salicaceae |  |  | .04 | .02 | 51 | 56-78 |
| Asteraceae | *Omalotheca* | *Omalotheca norvegica* | .02 | .02 | 35 | 49 |
|  | *Cirsium* | *Cirsium heterophyllum* | .00001 | .00002 | 1 | 46 |
| Orobanchaceae |  |  | .02 | .02 | 13 | 40-50 |
|  | *Euphrasia* |  | .01 | .02 | 4 | 42 |
|  |  | *Euphrasia wettsteinii* | .0006 | .001 | 2 | 42 |
|  | *Pedicularis* | *Pedicularis lapponica* | .003 | .004 | 6 | 50 |
|  | *Melampyrum* | *Melampyrum pratense* | .0002 | .0003 | 1 | 40 |
|  | *Bartsia* | *Bartsia alpina* | .00007 | .0001 | 2 | 44 |
| Equisetaceae | *Equisetum* |  | .02 | .02 | 20 | 11 |
| Primulaceae | *Trientalis* | *Trientalis europaea* | .01 | .01 | 20 | 42 |
| Ranunculaceae |  |  | .01 | .01 | 24 | 27-54 |
|  | *Ranunculus* |  | .007 | .007 | 21 | 27-54 |
|  | *Caltha* | *Caltha palustris* | .004 | .006 | 4 | 52 |
|  | *Trollius* | *Trollius europaeus* | .0005 | .001 | 2 | 49 |
| Caryophyllaceae |  |  | .01 | .02 | 5 | 23-66 |
|  | *Cerastium* |  | .01 | .02 | 4 | 48-59 |
|  | *Stellaria* |  | .00003 | .00005 | 1 | 54-55 |
|  |  | *Stellaria borealis* | .00002 | .00004 | 1 | 54 |
| Violaceae | *Viola* | *Viola biflora* | .003 | .005 | 5 | 51-52 |
| Onagraceae | *Epilobium* |  | .002 | .003 | 2 | 53-65 |
| Rosaceae |  |  | .002 | .003 | 2 | 43-66 |
| Geraniaceae | *Geranium* | *Geranium sylvaticum* | .001 | .002 | 4 | 55 |
| Cyperaceae |  |  | .001 | .001 | 4 | 78-90 |
|  | *Carex* |  | .00000 | .00001 | 1 | 81-90 |
| Cupressaceae | *Juniperus* | *Juniperus communis* | .0008 | .002 | 1 | 40 |
| Campanulaceae | *Campanula* |  | .001 | .001 | 2 | 45-50 |
| Woodsiaceae | *Gymnocarpium* |  | .0004 | .001 | 1 | 39 |
| Juncaceae | *Juncus* |  | .0003 | .0003 | 7 | 50-56 |
|  |  | *Juncus trifidus* | .0002 | .0002 | 6 | 50 |
| Pyrolaceae | *Pyrola* | *Pyrola minor* | .00007 | .00014 | 1 | 41 |
| Apiaceae |  |  | .00004 | .00006 | 2 | 44-57 |
| *Avena* and *Vitis* (trap bite) | |  | .02 | .02 | 38 |  |
| Brypophytes |  |  | .006 | .01 | 11 |  |
| Above family level |  |  | .007 | .005 | 23 |  |
