# Supplementary material for: Arctic Small Rodents Have Diverse Diets and Flexible Food Selection
Source: PLoS One. 2013 Jun 27;8(6):e68128. doi: 10.1371/journal.pone.0068128 (PMC3694920; doi:10.1371/journal.pone.0068128)
Supplement: Table S3 — Diet of Tundra voles (n = 67), in meadow habitat at Komagelva and Vestre Jakobselva, Varanger peninsula, during summer and autumn 2007. Mean proportion with standard error and frequency of occurrence (percentage of individuals where taxa present). Abundance of taxa at species level is included in the genera, which are included in families. Column “length g-h” refers to the length of the DNA region amplified with primer pair g-h, based on Sønstebø et al. (2010). (DOCX) [file pone.0068128.s003.docx]

| Family | genus | species | mean | SE | frequency | length *g-h* |
| --- | --- | --- | --- | --- | --- | --- |
| Polygonaceae |  |  | .45 | .09 | 97 | 27-32 |
|  | *Rumex* |  | .38 | .09 | 96 | 31 |
|  | *Bistorta* | *Bistorta vivipara* | .05 | .04 | 30 | 32 |
| Salicaceae |  |  | .17 | .07 | 70 | 56-78 |
| Poaceae |  |  | .13 | .05 | 93 | 48-58 |
|  | *Calamagrostis* |  | .04 | .03 | 55 | 58 |
|  | *Avenella* | *Avenella flexuosa* | .03 | .02 | 52 | 53 |
|  | *Festuca* |  | .02 | .02 | 42 | 53 |
|  | *Phleum* | *Phleum alpinum* | .01 | .01 | 34 | 53 |
|  | *Deschampsia* | *Deschampsia cespitosa* | .01 | .004 | 31 | 53 |
|  | *Anthoxanthum* | *Anthoxanthum nipponicum* | .01 | .003 | 37 | 53 |
|  | *Poa* |  | .003 | .002 | 39 | 53 |
|  |  | *Poa alpina* | .0005 | .0007 | 13 | 53 |
| Ranunculaceae |  |  | .12 | .06 | 51 | 27-72 |
|  | *Ranunculus* |  | .12 | .06 | 49 | 27-54 |
|  | *Trollius* | *Trollius europaeaus* | .002 | .003 | 4 | 49 |
|  | *Caltha* | *Caltha palustris* | .0001 | .0002 | 1 | 52 |
| Asteraceae |  |  | .04 | .02 | 40 | 46-55 |
|  | *Cirsium* | *Cirsium heterophyllum* | .02 | .02 | 15 | 46 |
|  | *Comarum* | *Comarum palustre* | .004 | .008 | 3 | 52 |
|  | *Saussurea* | *Saussurea alpina* | .002 | .003 | 4 | 50 |
| Pinaceae | *Pinus* | *Pinus sylvestris* | .01 | .03 | 1 | 45 |
| Primulaceae | *Trientalis* | *Trientalis europaea* | .02 | .03 | 15 | 42 |
| Caryophyllaceae |  |  | .01 | .01 | 24 | 23-66 |
|  | *Cerastium* |  | .01 | .01 | 21 | 48-59 |
|  |  | *Cerastium fontanum* | .0005 | .001 | 1 | 48 |
|  | *Stellaria* |  | .001 | .001 | 18 | 54-55 |
|  |  | *Stellaria nemorum* | .001 | .001 | 12 | 55 |
|  |  | *Stellaria borealis* | .0003 | .001 | 3 | 54 |
| Violaceae | *Viola* |  | .01 | .008 | 27 | 51-55 |
|  |  | *Viola biflora* | .004 | .004 | 7 | 51-52 |
| Equisetaceae | *Equisetum* |  | .006 | .004 | 36 | 11 |
| Ericaceae |  |  | .005 | .009 | 27 | 45-55 |
|  | *Vaccinium* |  | .004 | .007 | 10 | 47-50 |
|  |  | *Vaccinium myrtillus* | .00004 | .00005 | 6 | 48 |
|  |  | *Vaccinium uliginosum* | .00001 | .00002 | 1 | 45 |
|  | *Empetrum* | *Empetrum nigrum* | .00006 | .00006 | 7 | 46 |
| Rosaceae |  |  | .005 | .008 | 12 | 43-66 |
|  | *Alchemilla* | *Alchemilla glomerulans* | .0003 | .0004 | 6 | 53 |
|  | *Sibbaldia* | *Sibbaldia procumbens* | .00001 | .00003 | 1 | 52 |
| Apiaceae |  |  | .004 | .004 | 9 | 44-57 |
| Geraniaceae | *Geranium* | *Geranium sylvaticum* | .0008 | .002 | 1 | 55 |
| Orobanchaceae |  |  | .001 | .002 | 4 | 40-50 |
|  | *Rhinanthus* | *Rhinanthus minor* | .0008 | .002 | 1 | 45 |
|  | *Bartsia* | *Bartsia alpina* | .00001 | .00002 | 1 | 44 |
| Cyperaceae | *Carex* |  | .0002 | .0004 | 4 | 81-90 |
| Betulaceae | *Betula* |  | .0001 | .0001 | 7 | 61 |
| Woodsiaceae | *Athyrium* |  | .00003 | .00006 | 1 |  |
| Rubiaceae | *Galium* |  | .00001 | .00002 | 1 | 32-42 |
| Cornaceae | *Chamaepericlymenum* | *Chamaepericlymenum suecicum* | .00001 | .00002 | 1 | 50 |
| Juncaceae | *Juncus* | *Juncus trifidus* | .00001 | .00001 | 1 | 50 |
| *Avena* and *Vitis* (trap bite) | |  | .02 | .03 | 36 |  |
| Bryophyte |  |  | .00005 | .00005 | 6 |  |
| Above family level |  |  | .0003 | .0004 | 7 |  |
