# Supplementary material for: Arctic Small Rodents Have Diverse Diets and Flexible Food Selection
Source: PLoS One. 2013 Jun 27;8(6):e68128. doi: 10.1371/journal.pone.0068128 (PMC3694920; doi:10.1371/journal.pone.0068128)
Supplement: Table S4 — Grey-sided vole (n = 81) selectivity at plant functional group level, based on compositional analysis comparing used (plant DNA in individuals diet) against available (biomass of grid where the individual was trapped). The table is read along the rows; “+” indicates that food item on a row was used more than that in a column, “−” that it was less used. Tripled sign indicates significant differences. “dec.” refers to deciduous. (DOCX) [file pone.0068128.s004.docx]

|  | dec. shrub | ericoid | forb | grass | hemiparasite | sedge |
| --- | --- | --- | --- | --- | --- | --- |
| dec. shrub | 0 | — | — | - | + | +++ |
| ericoid | +++ | 0 | — | +++ | +++ | +++ |
| forb | +++ | +++ | 0 | +++ | +++ | +++ |
| grass | + | — | — | 0 | +++ | +++ |
| hemiparasite | - | — | — | — | 0 | +++ |
| sedge | — | — | — | — | — | 0 |
