# Supplementary material for: Arctic Small Rodents Have Diverse Diets and Flexible Food Selection
Source: PLoS One. 2013 Jun 27;8(6):e68128. doi: 10.1371/journal.pone.0068128 (PMC3694920; doi:10.1371/journal.pone.0068128)
Supplement: Table S7 — Tundra vole (n = 66) selectivity at plant family level, based on compositional analysis comparing used (plant DNA in individuals diet) against available (biomass of grid where the individual was trapped). The table is read along the rows; “+” indicates that food item on a row was used more than that in a column, “−” that it was less used. Tripled sign indicates significant differences. Columns are labeled with abbreviated family names using only the first three letters, rows are labeled with full names in the same order. (DOCX) [file pone.0068128.s007.docx]

|  | Api | Ast | Bet | Car | Cyp | Eri | Pri | Oro | Poa | Pol | Ran | Ros | Sal | Vio |
| --- | --- | --- | --- | --- | --- | --- | --- | --- | --- | --- | --- | --- | --- | --- |
| Apiaceae | 0 | +++ | +++ | + | +++ | +++ | +++ | +++ | — | — | — | +++ | - | +++ |
| Asteraceae | — | 0 | +++ | - | +++ | +++ | + | +++ | — | — | — | +++ | — | + |
| Betulaceae | — | — | 0 | — | - | — | — | — | — | — | — | — | — | — |
| Caryophyllaceae | - | + | +++ | 0 | +++ | +++ | +++ | +++ | — | — | — | +++ | — | +++ |
| Cyperaceae | — | — | + | — | 0 | — | — | — | — | — | — | - | — | — |
| Ericaceae | — | — | +++ | — | +++ | 0 | — | - | — | — | — | + | — | - |
| Primulaceae | — | - | +++ | — | +++ | +++ | 0 | +++ | — | — | — | +++ | — | + |
| Orobanchaceae | — | — | +++ | — | +++ | + | — | 0 | — | — | — | + | — | - |
| Poaceae | +++ | +++ | +++ | +++ | +++ | +++ | +++ | +++ | 0 | — | + | +++ | +++ | +++ |
| Polygonaceae | +++ | +++ | +++ | +++ | +++ | +++ | +++ | +++ | +++ | 0 | +++ | +++ | +++ | +++ |
| Ranunculaceae | +++ | +++ | +++ | +++ | +++ | +++ | +++ | +++ | - | — | 0 | +++ | + | +++ |
| Rosaceae | — | — | +++ | — | + | - | — | - | — | — | — | 0 | — | — |
| Salicaceae | + | +++ | +++ | +++ | +++ | +++ | +++ | +++ | — | — | - | +++ | 0 | +++ |
| Violaceae | — | - | +++ | — | +++ | + | - | + | — | — | — | +++ | — | 0 |
